# Bis(2-bromo­pyridinium) hexa­bromido­stannate(IV)

**DOI:** 10.1107/S1600536808012129

**Published:** 2008-05-03

**Authors:** Basem Fares Ali, Rawhi H. Al-Far, Salim F. Haddad

**Affiliations:** a Department of Chemistry, Al al-Bayt University, Mafraq 25113, Jordan; b Faculty of Information Technology and Science, Al-Balqa’a Applied University, Salt, Jordan; c Department of Chemistry, The University of Jordan, Amman, Jordan

## Abstract

The asymmetric unit of the title compound, (C_5_H_5_BrN)_2_[SnBr_6_], contains one cation and one half-anion. The [SnBr_6_]^2−^ anion is located on an inversion center and forms a quasi-regular octa­hedral arrangement. The crystal structure consists of two-dimensional supra­molecular layers assembled *via* hydrogen-bonding inter­actions of N—H⋯Br—Sn [*D*⋯*A* = 3.375 (13)–3.562 (13) Å and *D*—H⋯*A* = 127–142°, along with C—Br⋯Br synthons [3.667 (2) and 3.778 (3) Å]. These layers are parallel to the *bc* plane and built up from anions inter­acting extensively with the six surrounding cations.

## Related literature

The title salt is isomorphous with the Te analogue (Fernandes *et al.*, 2004 ▶). For related literature, see: Al-Far & Ali (2007 ▶); Ali, Al-Far & Al-Sou’od (2007 ▶); Ali & Al-Far (2007 ▶); Ali, Al-Far & Ng (2007 ▶); Allen *et al.* (1987 ▶); Aruta *et al.* (2005 ▶); Hill (1998 ▶); Kagan *et al.* (1999 ▶); Knutson *et al.* (2005 ▶); Raptopoulou *et al.* (2002 ▶); Tudela & Khan (1991 ▶); Willey *et al.* (1998 ▶).
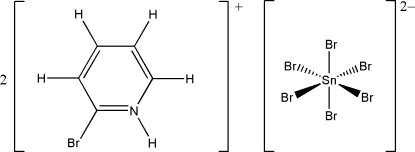


## Experimental

### 

#### Crystal data

(C_5_H_5_BrN)_2_[SnBr_6_]
*M* *_r_* = 916.12
Triclinic, 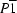

*a* = 7.4037 (15) Å
*b* = 8.3393 (17) Å
*c* = 9.4302 (19) Å
α = 73.14 (3)°
β = 67.98 (3)°
γ = 82.44 (3)°
*V* = 516.4 (2) Å^3^
*Z* = 1
Mo *K*α radiation
μ = 16.71 mm^−1^
*T* = 293 (2) K
0.16 × 0.13 × 0.08 mm

#### Data collection

Bruker–Siemens SMART APEX diffractometer
Absorption correction: multi-scan (*SADABS*; Bruker, 2007 ▶) *T*_min_ = 0.058, *T*_max_ = 0.261
2266 measured reflections
1807 independent reflections
1308 reflections with *I* > 2σ(*I*)
*R*_int_ = 0.091

#### Refinement

*R*[*F* ^2^ > 2σ(*F* ^2^)] = 0.068
*wR*(*F* ^2^) = 0.178
*S* = 1.02
1807 reflections
67 parameters
H-atom parameters constrained
Δρ_max_ = 3.31 e Å^−3^
Δρ_min_ = −1.87 e Å^−3^

### 

Data collection: *SMART* (Bruker, 2006 ▶); cell refinement: *SAINT-Plus* (Bruker, 2006 ▶); data reduction: *SAINT-Plus*; program(s) used to solve structure: *XS* in *SHELXTL* (Sheldrick, 2008 ▶); program(s) used to refine structure: *XL* in *SHELXTL*; molecular graphics: *XP* in *SHELXTL*; software used to prepare material for publication: *XCIF* in *SHELXTL*.

## Supplementary Material

Crystal structure: contains datablocks I, global. DOI: 10.1107/S1600536808012129/bx2139sup1.cif

Structure factors: contains datablocks I. DOI: 10.1107/S1600536808012129/bx2139Isup2.hkl

Additional supplementary materials:  crystallographic information; 3D view; checkCIF report

## Figures and Tables

**Table:** 

Sn1—Br3	2.5939 (15)
Sn1—Br1	2.6027 (15)
Sn1—Br4	2.6174 (17)

**Table:** 

Br3—Sn1—Br1	89.06 (5)
Br3^i^—Sn1—Br1	90.94 (5)
Br3—Sn1—Br4^i^	89.43 (6)
Br1—Sn1—Br4^i^	90.21 (5)
Br3—Sn1—Br4	90.57 (6)
Br1—Sn1—Br4	89.79 (5)

Symmetry code: (i) 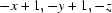.

**Table 2:** Hydrogen-bond geometry (Å, °)

*D*—H⋯*A*	*D*—H	H⋯*A*	*D*⋯*A*	*D*—H⋯*A*
N1—H1⋯Br4^ii^	0.86	2.65	3.375 (13)	142
N1—H1⋯Br1^iii^	0.86	2.98	3.562 (13)	127

Symmetry codes: (ii) 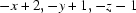; (iii) 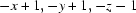.

**Fig. 1.**
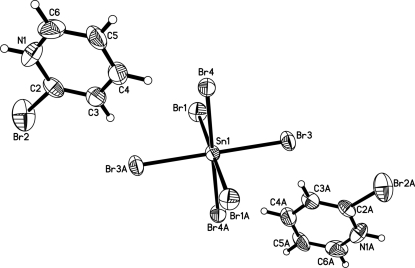
A view of the asymmetric unit of (I) with the atom-numbering scheme. Displacement ellipsoids are drawn at the 50% probability level.

**Fig. 2.**
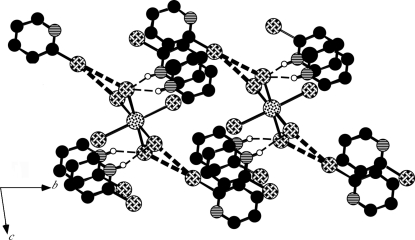
A packing diagram of (I). Hydrogen bonds (dashed lines) and Br···Br interactions (thick dashed lines) are shown for (SnBr6)2- anions and six surrounding cations.

## supplementary crystallographic information

### Comment

Noncovalent interactions play an important role in organizing structural units in both natural and artificial systems. Hybrid organic-inorganic compounds are of great interest owing to their ionic, electrical, magnetic and optical properties (Hill, 1998; Kagan *et al.*, 1999; Raptopoulou *et al.*, 2002). Tin metal-halo based hybrids are of particular interest as being materials with interesting optical and magnetic properties (Aruta *et al.*, 2005; Knutson *et al.*, 2005; Kagan *et al.*, 1999). We are currently carrying out studies about lattice including different types of intermolecular interactions (aryl···aryl, *X*···*X*, *X*···aryl and *X*···H). Within our research of hybrid compounds containing tin metal (Al-Far & Ali 2007; Ali, Al-Far & Al-Sou'od, 2007; Ali & Al-Far, 2007; Ali, Al-Far & Ng, 2007), the crystal structure of the title salt, (I), has been investigated.

The asymmetric unit of (I) contains one cation and one-half anion (Fig. 1). The whole (2-Br—C_5_H_5_N)_2_[SnBr_6_] geometry is generated through an inversion center with tin being lying on the special crystallographic position of (1/2, 1/2, 0). The (SnBr_6_)^2-^ anion forms a quasi-octahedral geometry (Table 1), with the Sn—Br bond lengths are almost invariant. These lengths are in accordance with tin-bromide distances reported for (SnBr_6_)^2-^ anion containing compounds (Willey *et al.*,1998; Tudela & Khan 1991; Al-Far & Ali 2007; Ali, Al-Far & Al-Sou'od, 2007; Ali & Al-Far, 2007; Ali, Al-Far & Ng, 2007). Bond lengths and angles within the cation are as expected (Allen *et al.*, 1987).

The packing of the structure (Fig. 2) can be described as layers of alternating anions and cations parallel to *bc* plane. In these layers each (SnBr_6_)^2-^ anion is interacting with six cations *via* two N—H···Br interactions (Table 2) and the symmetry related ones along with two Br···Br interactions and symmetry related ones [Br2···Br4and Br2···Br1of 3.6666 (23) and 3.7779 (29) Å, respectively; Fig. 2].

The N—H···N interactions along with C—Br···Br synthons are potential building blocks for this stable supramolecular lattice. The stability of this lattice is evident in the isostructurality with the reported Te analogue (Fernandes *et al.*, 2004).

### Experimental

Warm solution of Sn metal (1.0 mmol) dissolved in absolute ethanol (10 ml) and HBr (60%, 5 ml), was added dropwise to a stirred hot solution of 2-bromopyridine (2 mmol) dissolved in ethanol (10 ml). The mixture was then treated with liquid Br_2_ (2 ml) and refluxed for 3/2 h. The resulting mixture was then filtered off, and allowed to stand undisturbed at room temperature. The salt crystallized over 1 d as nice yellow block crystals (yield: 83%).

### Refinement

H atoms were positioned geometrically, with N—H = 0.86 Å (for NH) and C—H = 0.93 Å for aromatic H, and constrained to ride on their parent atoms, with *U*_iso_(H) = 1.2*U*_eq_(C, N).

### Crystal data

**Table:**

(C_5_H_5_BrN)_2_[SnBr_6_]	*Z* = 1
*M**_r_* = 916.12	*F*_000_ = 414
Triclinic, *P*1	*D*_x_ = 2.946 Mg m^−^^3^
Hall symbol: -P 1	Mo *K*α radiation λ = 0.71073 Å
*a* = 7.4037 (15) Å	Cell parameters from 255 reflections
*b* = 8.3393 (17) Å	θ = 2.1–27.7º
*c* = 9.4302 (19) Å	µ = 16.71 mm^−^^1^
α = 73.14 (3)º	*T* = 293 (2) K
β = 67.98 (3)º	Block, yellow
γ = 82.44 (3)º	0.16 × 0.13 × 0.08 mm
*V* = 516.4 (2) Å^3^	

### Data collection

**Table:**

Bruker–Siemens SMART APEX diffractometer	1807 independent reflections
Radiation source: fine-focus sealed tube	1308 reflections with *I* > 2σ(*I*)
Monochromator: graphite	*R*_int_ = 0.091
Detector resolution: 8.3 pixels mm^-1^	θ_max_ = 25.0º
*T* = 293(2) K	θ_min_ = 2.4º
ω scans	*h* = −1→8
Absorption correction: multi-scan(SADABS; Bruker, 2007)	*k* = −9→9
*T*_min_ = 0.058, *T*_max_ = 0.261	*l* = −10→11
2266 measured reflections	

### Refinement

**Table:**

Refinement on *F*^2^	Secondary atom site location: difference Fourier map
Least-squares matrix: full	Hydrogen site location: inferred from neighbouring sites
*R*[*F*^2^ > 2σ(*F*^2^)] = 0.068	H-atom parameters constrained
*wR*(*F*^2^) = 0.178	*w* = 1/[σ^2^(*F*_o_^2^) + (0.1076*P*)^2^ + 1.002*P*] where *P* = (*F*_o_^2^ + 2*F*_c_^2^)/3
*S* = 1.02	(Δ/σ)_max_ < 0.001
1807 reflections	Δρ_max_ = 3.31 e Å^−^^3^
67 parameters	Δρ_min_ = −1.87 e Å^−^^3^
Primary atom site location: structure-invariant direct methods	Extinction correction: none

### Special details

**Table:**

Geometry. All e.s.d.'s (except the e.s.d. in the dihedral angle between two l.s. planes) are estimated using the full covariance matrix. The cell e.s.d.'s are taken into account individually in the estimation of e.s.d.'s in distances, angles and torsion angles; correlations between e.s.d.'s in cell parameters are only used when they are defined by crystal symmetry. An approximate (isotropic) treatment of cell e.s.d.'s is used for estimating e.s.d.'s involving l.s. planes.
Refinement. Refinement of *F*^2^ against ALL reflections. The weighted *R*-factor *wR* and goodness of fit *S* are based on *F*^2^, conventional *R*-factors *R* are based on *F*, with *F* set to zero for negative *F*^2^. The threshold expression of *F*^2^ > σ(*F*^2^) is used only for calculating *R*-factors(gt) *etc*. and is not relevant to the choice of reflections for refinement. *R*-factors based on *F*^2^ are statistically about twice as large as those based on *F*, and *R*- factors based on ALL data will be even larger.

### Fractional atomic coordinates and isotropic or equivalent isotropic displacement parameters (Å^2^)

**Table:**

	*x*	*y*	*z*	*U*_iso_*/*U*_eq_	
Sn1	0.5000	0.5000	0.0000	0.0271 (4)	
Br4	0.80819 (19)	0.51691 (17)	−0.25738 (17)	0.0365 (4)	
Br3	0.5601 (2)	0.17999 (16)	0.09614 (18)	0.0394 (4)	
Br1	0.2879 (2)	0.43877 (19)	−0.14375 (19)	0.0426 (4)	
Br2	0.6761 (3)	0.2489 (2)	−0.4484 (2)	0.0674 (6)	
C2	0.7936 (19)	0.1051 (18)	−0.5793 (18)	0.038 (3)*	
N1	0.9316 (17)	0.1687 (17)	−0.7225 (17)	0.049 (3)*	
H1	0.9575	0.2732	−0.7499	0.059*	
C3	0.751 (2)	−0.0578 (18)	−0.5321 (19)	0.042 (3)*	
H3	0.6604	−0.1039	−0.4324	0.051*	
C4	0.845 (2)	−0.155 (2)	−0.6371 (19)	0.047 (4)*	
H4	0.8119	−0.2665	−0.6094	0.057*	
C5	0.990 (2)	−0.090 (2)	−0.784 (2)	0.051 (4)*	
H5	1.0572	−0.1560	−0.8526	0.061*	
C6	1.028 (3)	0.077 (2)	−0.822 (2)	0.060 (5)*	
H6	1.1225	0.1254	−0.9183	0.072*	

### Atomic displacement parameters (Å^2^)

**Table:**

	*U*^11^	*U*^22^	*U*^33^	*U*^12^	*U*^13^	*U*^23^
Sn1	0.0304 (6)	0.0249 (6)	0.0213 (7)	0.0021 (5)	−0.0058 (5)	−0.0048 (5)
Br4	0.0387 (7)	0.0322 (7)	0.0294 (8)	−0.0003 (5)	−0.0011 (6)	−0.0094 (6)
Br3	0.0462 (8)	0.0258 (7)	0.0340 (9)	0.0066 (6)	−0.0067 (6)	−0.0034 (6)
Br1	0.0491 (8)	0.0446 (8)	0.0391 (9)	−0.0002 (6)	−0.0226 (7)	−0.0094 (7)
Br2	0.1089 (15)	0.0522 (10)	0.0491 (11)	0.0059 (10)	−0.0295 (11)	−0.0268 (9)

### Geometric parameters (Å, °)

**Table:**

Sn1—Br3	2.5939 (15)	N1—C6	1.32 (2)
Sn1—Br3^i^	2.5939 (15)	N1—H1	0.8600
Sn1—Br1	2.6027 (15)	C3—C4	1.39 (2)
Sn1—Br1^i^	2.6027 (15)	C3—H3	0.9300
Sn1—Br4^i^	2.6174 (17)	C4—C5	1.40 (2)
Sn1—Br4	2.6174 (17)	C4—H4	0.9300
Br2—C2	1.870 (15)	C5—C6	1.37 (2)
C2—C3	1.34 (2)	C5—H5	0.9300
C2—N1	1.357 (19)	C6—H6	0.9300
			
Br3—Sn1—Br3^i^	180.0	N1—C2—Br2	117.9 (11)
Br3—Sn1—Br1	89.06 (5)	C6—N1—C2	122.7 (14)
Br3^i^—Sn1—Br1	90.94 (5)	C6—N1—H1	118.6
Br3—Sn1—Br1^i^	90.94 (5)	C2—N1—H1	118.6
Br3^i^—Sn1—Br1^i^	89.06 (5)	C2—C3—C4	117.8 (15)
Br1—Sn1—Br1^i^	180.00 (5)	C2—C3—H3	121.1
Br3—Sn1—Br4^i^	89.43 (6)	C4—C3—H3	121.1
Br3^i^—Sn1—Br4^i^	90.57 (6)	C3—C4—C5	121.7 (15)
Br1—Sn1—Br4^i^	90.21 (5)	C3—C4—H4	119.1
Br1^i^—Sn1—Br4^i^	89.79 (5)	C5—C4—H4	119.1
Br3—Sn1—Br4	90.57 (6)	C6—C5—C4	116.8 (17)
Br3^i^—Sn1—Br4	89.43 (6)	C6—C5—H5	121.6
Br1—Sn1—Br4	89.79 (5)	C4—C5—H5	121.6
Br1^i^—Sn1—Br4	90.21 (5)	N1—C6—C5	120.6 (17)
Br4^i^—Sn1—Br4	180.0	N1—C6—H6	119.7
C3—C2—N1	120.3 (15)	C5—C6—H6	119.7
C3—C2—Br2	121.8 (12)		
			
C3—C2—N1—C6	1(2)	C2—C3—C4—C5	4(2)
Br2—C2—N1—C6	177.7 (12)	C3—C4—C5—C6	−3(2)
N1—C2—C3—C4	−3(2)	C2—N1—C6—C5	1(3)
Br2—C2—C3—C4	−179.9 (11)	C4—C5—C6—N1	0(3)

Symmetry codes: (i) −*x*+1, −*y*+1, −*z*.

### Hydrogen-bond geometry (Å, °)

**Table:**

*D*—H···*A*	*D*—H	H···*A*	*D*···*A*	*D*—H···*A*
N1—H1···Br4^ii^	0.86	2.65	3.375 (13)	142
N1—H1···Br1^iii^	0.86	2.98	3.562 (13)	127

Symmetry codes: (ii) −*x*+2, −*y*+1, −*z*−1; (iii) −*x*+1, −*y*+1, −*z*−1.
